# Baseline Levels of Serum Progesterone and the First Trimester Pregnancy Outcome in Women with Threatened Abortion: A Retrospective Cohort Study

**DOI:** 10.1155/2020/8780253

**Published:** 2020-03-02

**Authors:** Yongshi Deng, Chi Chen, Siyun Chen, Guanyan Mai, Xiuping Liao, He Tian, Wenli Liu, Shuling Ji, Ying Liu, Jie Gao, Songping Luo

**Affiliations:** ^1^The First Clinical Medical College, Guangzhou University of Chinese Medicine, Guangzhou 510000, China; ^2^Department of Immunology and Microbiology, Guiyang College of Traditional Chinese Medicine, Guiyang 550000, China; ^3^Department of Obstetrics and Gynecology, The First Affiliated Hospital of Guangzhou University of Chinese Medicine, Guangzhou 510000, China

## Abstract

**Objective:**

The relationship between serum progesterone and the first trimester pregnancy outcome of threatened abortion is still controversial. Therefore, we aimed to further study the association between these two parameters.

**Methods:**

The present study is an observational retrospective cohort study. A total of 726 participants who had threatened abortion from a hospital in Guangdong, China, were included in this study from 17th August 2011 to 30th October 2018. The exposure variable and the outcome variable were serum progesterone measured at baseline and early pregnancy outcome, respectively. Covariates involved in this study included patients' basic demographics, obstetric history, and clinical information.

**Results:**

A negative association and a saturation effect were detected between serum progesterone and the first trimester pregnancy outcome. When progesterone <90.62 nmol/L, an increase in 1 nmol/L of serum progesterone was associated with 3% decrease of the risk of miscarriage (OR: 0.97, 95% CI: 0.95-0.98).

**Conclusion:**

There was a greater risk of abortion when the serum progesterone level was less than 90.62 nmol/L. Our findings can better assist the clinician in understanding patients' conditions and making medical decisions.

## 1. Introduction

Threatened abortion is defined as vaginal bleeding through a closed cervical os, with or without abdominal pain, during the first 20 weeks of pregnancy [1]. Threatened abortion may be one of the most common gynecological conditions and occurs in 25% of pregnancies [2]. Unfortunately, about 10% to 20% of the women who have threatened abortion may experience a miscarriage [2], which may cause depression, anxiety, and even posttraumatic stress symptoms [3].

Progesterone is a sex steroid which plays an important role in women's menstrual cycles and pregnancy [4, 5]. It can induce endometrium change into the secretory phase, preparing for fertilized egg implantation [4]. In terms of immunity, it can regulate the balance of cytokines and influence the activity of natural killer cells [5, 6]. It suppresses uterine contraction, by enhancing nitric oxide production in the lining of the uterus [7, 8]. Furthermore, progesterone may reduce the spiral arteries' pulsatility index and resistance index; thus, it can increase uteroplacental circulation [9].

Several studies showed that low serum progesterone was associated with abortion [10–14]. Hence, exogenous progesterone supplements were often used to treat threatened abortion, especially in China [15]. However, the efficacy of progesterone is controversial [16–19], and the applicable standard is still vague [15, 16]. In this study, we aim to further analyse the relationship between serum progesterone and the first trimester pregnancy outcome.

## 2. Materials and Methods

### 2.1. Study Participants

**Figure 1:**
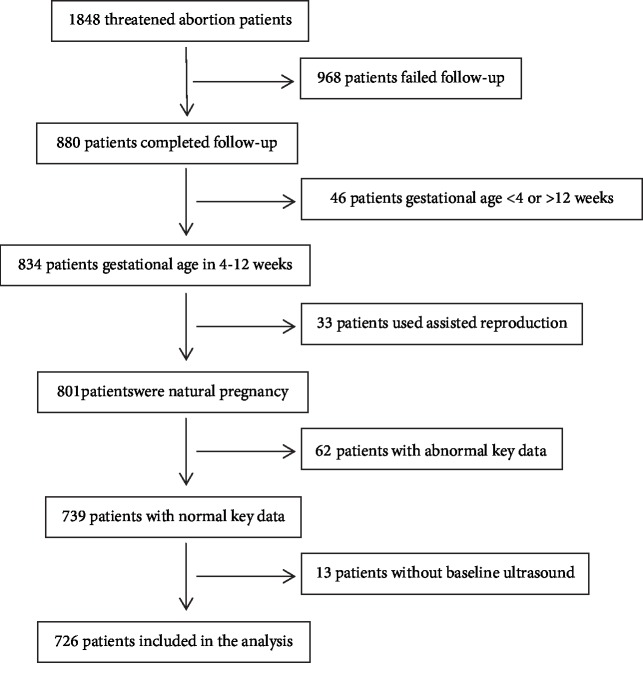
Flow chart.

This study is a retrospective cohort study conducted at the First Affiliated Hospital of Guangzhou University of Chinese Medicine. The clinical information of women with threatened abortion from the hospital's electronic medical record system was collected from 17th August 2011 to 30th October 2018. There was no sample size calculation. For convenience, the sample size of this study was based on previous similar studies [20–24]. A total of 1848 threatened abortion patients were initially included. After screening, 726 cases were available for data analysis (see Figure 1 for details).

The inclusion criteria were intrauterine pregnancy with pregnancy-related vaginal bleeding [1] between 4 and 12 weeks of gestation. For all patients, the gestational age was calculated from the date of last menstruation.

The exclusion criteria were as follows: (1) Patients developed vaginal bleeding because of other reasons rather than threatened abortion. (2) Patients who were pregnant through assisted reproductive technology. (3) Patients were diagnosed as missed abortion or inevitable miscarriage at admission. (4) Patients eventually were diagnosed with ectopic pregnancy. (5) Patients without baseline ultrasound. (6) Patients who had been using progesterone before inclusion in the study.

### 2.2. Exposure Variable

The first measurement of serum progesterone was made on the second day after admission. Doctors determined the next hormone measurement time based on the patient's condition, normally between 3 and 7 days after the first measurement.

### 2.3. Outcome Variable

Our primary outcome was spontaneous miscarriage at the first trimester, including inevitable abortion, incomplete abortion, complete abortion, and missed abortion. The diagnosis of miscarriage was made by a clinician; they must be based on the patient's symptoms, signs, serum *β*-HCG, serum progesterone, and ultrasound. The follow-up consultation by telephone was made at 13 weeks of gestation.

### 2.4. Covariates

The selection of covariates in this study was mainly based on previous studies which researched the relationship between progesterone and pregnancy outcome [10–14, 17–22]. Therefore, patients' basic demographics (age, height, weight, smoking history, drinking history, and marital status), obstetric history (times of gestity, parity, and abortion), and clinical information (gestational week, progesterone, *β*-HCG, medical history, and use of progesterone) were collected. We compiled patients' basic demographics, obstetric history, and partial clinical information at the time of enrollment. The first measurement of serum *β*-HCG was on the second day after admission, and the measurement interval was 3-7 days. Log10 transformation of *β*-HCG was used to reduce the skewness of its data distribution. We obtained medication information through medical records.

### 2.5. Treatment

Progestogen preparations used in our hospital included dydrogesterone tablets, progesterone injections, and progesterone capsules. The type and dose of progestogen were selected based on the patient's symptoms, auxiliary examinations, and medical history, followed the Luteal Support and Progesterone Supplementation Consensus of the Chinese Medical Association Reproductive Medicine Branch [15].

### 2.6. Statistical Methods

Categorical variables were shown in frequency or percentage. Continuous variables were expressed in mean ± standard deviation (SD) when they had normal distribution. Continuous variables were expressed in median with quartile when they had skewed distribution. To investigate whether baseline levels of serum progesterone was correlated with the first trimester pregnancy outcome in threatened abortion women, our statistical analysis included three main steps.

*Step 1*: we used chi-square test (categorical variables), Student *t*-test (normal distribution), or Mann-Whitney *U* test (skewed distribution) to calculate the difference of baseline characteristics for patients with different pregnancy outcome in Table 1. The first measurement of serum progesterone and *β*-HCG was reported.

*Step 2*: binary logistic regression and marginal structural model were used for multivariate analysis. Three models were constructed in Table 2: model 1, binary logistic regression without adjusted covariates, used the first measurement of serum progesterone; model 2, binary logistic regression adjusted all covariates presented in Table 1, used the first measurement of serum progesterone and *β*-HCG; and model 3, marginal structural model adjusted all covariates in Table 1, used the serial measurements of serum progesterone and *β*-HCG.

To ensure the robustness of data analysis, we made a sensitivity analysis. (1) Serum progesterone changed over time [11] and had two-way causality with progesterone therapy. Therefore, we used marginal structural model for sensitivity analysis. (2) In order to verify the results of progesterone as the continuous variable, and to examine the possibility of nonlinearity, we converted basal progesterone into a categorical variable according to the tertile; we also performed an interaction test and calculated the *P* for trend.

**Figure 2:**
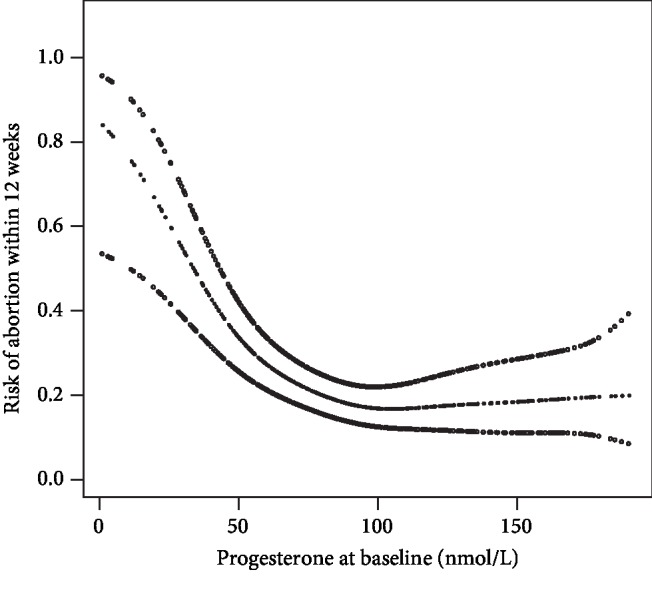
Smooth curve of progesterone and abortion.

*Step 3*: to research the nonlinearity relationship of basal progesterone and early pregnancy outcomes, a binary logistic regression and smooth curve fitting (penalized spline method) were conducted in Table 3 and Figure 2. If nonlinearity was detected, we used both binary logistic regression and two-piecewise binary logistic regression to fit the association and selected the best fit model based on *P* for the log-likelihood ratio test. Then, we calculated the inflection point using recursive algorithm and constructed a two-piecewise linear regression model on both sides of the inflection point.

All the analyses were performed with the statistical software packages R (http://www.R-project.org, The R Foundation) and EmpowerStats (http://empowerstats.com, X&Y Solutions, Inc., Boston, MA). *P* values less than 0.05 (two-sided) were considered statistically significant.

### 2.7. Ethics

This study was approved by the ethical review board of the First Affiliated Hospital of Guangzhou University of Chinese Medicine.

## 3. Results

### 3.1. Study Participants

After screening, according to inclusion criteria and exclusion criteria (see Figure 1 for details), 726 of participants were finally adopted in statistics, including 28.65% (208 of 726 patients) miscarriage and 71.35% (518 of 726 patients) ongoing pregnancy within 12 weeks of gestation.

As shown in Table 1, compared with continue pregnancy patients, participants in the miscarriage group had higher maternal age, higher gestity, lower basal progesterone, lower basal *β*-HCG (log10), lower embryo visualized rate, and lower cardiac movement visualized rate.

### 3.2. Multivariate Analysis

In this research, we constructed three models to observe the relationship between serum progesterone and early pregnancy outcome. The odds ratio (OR) and 95% confidence intervals (CI) are listed in Table 2. The model-based effect size can explain the difference between every one unit of serum progesterone and the association with the risk of miscarriage.

In model 1 (nonadjusted model), an increase in 1 nmol/L of basal serum progesterone was associated with 2% decrease of the risk of miscarriage (OR: 0.98, 95% CI: 0.98-0.99). In model 2 (fully adjusted model), the basal progesterone increased by 1 nmol/L and the risk of miscarriage decreased by 1% (OR: 0.99, 95% CI: 0.98-0.99). In model 3 (marginal structural model), 1 nmol/L increase of serial progesterone was also associated with 1% decrease of the risk of miscarriage (HR: 0.99, 95% CI: 0.98-0.99). This suggested that the results were stable and baseline serum progesterone can be used as an exposure factor to discuss its relationship with pregnancy outcomes. Besides, we found that the trend of the ORs in different basal progesterone groups was nonequidistant, which meant nonlinearity existed.

### 3.3. Nonlinearity Analysis

By using binary logistic regression and the penalty curve method, we found the nonlinear relationship between baseline serum progesterone and the risk of abortion to be within 12 weeks (Table 3 and Figure 2).

By recursive algorithm, we got the inflection point of 90.62 nmol/L. When basal serum progesterone was <90.62 nmol/L, an increase in 1 nmol/L of progesterone was associated with 3% decrease of the risk of miscarriage (OR: 0.97, 95% CI: 0.95-0.98). When basal serum progesterone was >90.62 nmol/L, the increase in progesterone did not affect the pregnancy outcome (OR: 1.01, 95% CI: 0.99-1.02). This result indicated that there was a saturation effect on the relationship between baseline serum progesterone and early pregnancy outcome. When basal serum progesterone was <90.62 nmol/L, the risk of miscarriage decreased with the rise of progesterone. When basal serum progesterone was >90.62 nmol/L, the risk of miscarriage would not decrease even if progesterone was elevated.

## 4. Discussion

Our study was aimed at investigating the relationship between serum progesterone levels and pregnancy outcome within 12 weeks of gestation in threatened abortion women. The results showed that basal serum progesterone had a negative association with the risk of the first trimester abortion; even the effects of time and progesterone therapy were considered. In addition, we found a saturation effect between basal serum progesterone and the first trimester abortion risk. Their negative association could only be observed when *P* < 90.62 nmol/L.

Markers such as *β*-HCG, CA125, ultrasound parameters, and progesterone are related to pregnancy outcome. However, some of them have their own disadvantages in assessing clinical condition. With serum *β*-HCG doubling approximately every 2 days and having individual differences [4], repeated measurements are needed to assess the embryo condition. CA125 is not a common measurement for threatened abortion patients, and studies about CA125 as a prediction of pregnancy outcomes are still controversial [23–25]. Additional CA125 detection may increase medical expenses. Ultrasound is commonly used to assess the condition of an embryo [26, 27]. However, parameters such as gestational sac diameter (GSD), yolk sac diameter (YSD), crown-rump length (CRL), and heart rate (HR) are difficult to obtain before the pregnancy sac is seen. Serum progesterone is a commonly used detection in threatened abortion women, its fluctuation is relatively small (compared with HCG), and the measurement is not limited by the gestational age. As far as we know, most studies suggested that serum progesterone levels were associated with pregnancy outcome, especially in the first trimester. Serum progesterone in abortion patients was generally lower than that in continuing pregnancy [10–14]. In our study, a negative correlation between serum progesterone and early pregnancy failure was further described. The risk of abortion decreased with the rise of serum progesterone.

In recent years, researches on the relationship between serum progesterone and pregnancy outcomes focused mainly on predictive models. Osmanağaoğlu et al. studied the predictive effects of progesterone, *β*-HCG, and CA125 for abortion. They found that using progesterone < 15 ng/mL (around 47.7 nmol/L) (sensitivity = 91%, specificity = 89%, PPV = 59%, and NPV = 98%) or *β* − HCG < 20 ng/mL (sensitivity = 91%, specificity = 82%, PPV = 46%, and NPV = 98%) alone could better predict abortion than using them together (sensitivity = 81%, specificity = 99%, PPV = 94%, and NPV = 97%) [24]. A study about the single measurement of serum progesterone was carried out; it showed that 32.7 ng/mL (around 103.99 nmol/L) (sensitivity = 90%, specificity = 92%, PPV = 97%, and NPV = 75%) was the cut-off value of early pregnancy failure [20]. Al Mohamady et al. used progesterone and CA125 to predict pregnancy outcomes in patients with threatened miscarriage. The result showed both of them were valid predictions. The cut-off value of progesterone was 11.5 ng/mL (around 36.57 nmol/L) (sensitivity = 97.5%, specificity = 100%, overall accuracy = 99.8%) [23]. Ku et al. used progesterone, fetal heart, and BMI to predict the outcome of spontaneous miscarriage. The ROC analysis showed a cut point of about 35 nmol/L progesterone (sensitivity = 77%, specificity = 88%, PPV = 68%, and NPV = 92%) as the independent prediction [21]. Lek et al. had a verification experiment on this cut point; the result showed that 35 nmol/L was accurate and reproducible [22]. These studies showed that basal progesterone could be used as a prognostic marker of threatened abortion.

Although progesterone therapy is often used in patients with threatened abortion, the therapeutic effect is controversial. The European Progestin Club proposed that dydrogesterone can reduce the rate of spontaneous miscarriage in threatened abortion women [16]. A meta-analysis (combined with seven studies, a total of 696 participants) suggested that progesterone would decrease the miscarriage rate (RR 0.64, 95% CI 0.47 to 0.87) [17]. At the same time, another meta-analysis (combined with fourteen trials, a total of 2158 women) showed there was no statistically different abortion rate between women using progesterone therapy or not (Peto OR 0.99, 95% CI 0.78 to 1.24). In addition, the subgroup analysis (combined four trials, a total of 225 women) suggested that progesterone treatment might decrease the abortion rate of women with recurrent miscarriages (Peto OR 0.39, 95% CI 0.21 to 0.72) [19]. A multicenter, randomized, double-blind, placebo controlled trial in the United Kingdom indicated there was no significant increase in threatened abortion women's live birth rate in the progesterone group compared to the placebo group (75% vs. 72%, RR 1.03, 95% CI 1.00 to 1.07, *P* = 0.08) [18]. Based on our study, baseline serum progesterone had an impact on pregnancy outcomes within a certain range. Previous studies about progesterone treatment did not perform relevant analysis based on the patient's progesterone level, which may affect the results of the studies.

All of the above evidence showed that serum progesterone was closely related to pregnancy outcomes, but the effectiveness of progesterone therapy is controversial. Our study found a saturation effect between serum progesterone and early pregnancy failure in threatened abortion patients. Only when *P* < 90.62 nmol/L did abortion risk have negative association with serum progesterone. This prompted us to think that progesterone therapy may need more precise indications, especially in terms of serum progesterone values. A classic series study by Csapo et al. showed that patients who had ovariectomy or luteectomy at about 7 weeks of pregnancy would have progesterone decrease and abortion [28]. Luteectomized patients who had progesterone therapy sustained normal pregnancy [29]. Hensleigh and Fainstat conducted a study on corpus luteum dysfunction women with threatened or recurrent abortion. In 9/11 threatened abortion women who received progesterone therapy, serum progesterone was corrected and no abortion occurred [30]. Palagiano et al. conducted a prospective study on threatened abortion women with corpus luteum dysfunction. Compared to placebo, vaginal progesterone could reduce pain, uterine contractions, and abortion rate (*P* < 0.05) [31]. These studies suggested that women with low serum progesterone could benefit from progesterone therapy. In China, threatened abortion patients with luteal dysfunction are recommended progesterone replacement [32]. However, the indications for progesterone therapy remain unclear. At present, there are no definitive values to diagnose progesterone deficiency [5]. Even considering luteal dysfunction as a replacement indication, the diagnosis of luteal dysfunction is controversial [33]. Therefore, large-scale, multicenter studies are needed to further evaluate the indication and effect of progesterone therapy.

Compared with previous studies, our research has the following progress. First of all, we found the inflection point of the saturation effect between baseline serum progesterone and first trimester abortion, which can better assist the clinician in understanding patients' conditions and making medical decisions. Secondly, we proposed to further clarify indications for progesterone therapy in threatened abortion patients. Moreover, considering the effect of time and progesterone therapy on serum progesterone, we used a marginal structural model to demonstrate the reliability of our results.

## 5. Limitation

Our study had some limitations. Since our study was aimed at natural pregnancy patients with threatened abortion, the results of this study are only applicable to this population. Secondly, this study discussed the relationship between serum progesterone and the first trimester pregnancy outcome, and the results may not be applicable to delivery outcome. Thirdly, due to the limitation of the sample size, there was no subgroup analysis for different kinds of progesterone supplement.

## 6. Conclusions

In conclusion, our study showed a negative association and a saturation effect between baseline serum progesterone and the first trimester pregnancy outcome in threatened abortion women. This provided an idea that serum progesterone values may be considered as a reference for progesterone supplement. Further studies are needed to verify whether serum progesterone is a better indication for progesterone replacement.

## Figures and Tables

**Table 1:** Baseline characteristics of participants.

Pregnancy outcome	Continue pregnancy	Miscarriage	*P* value
*N* (%)	518 (71.35%)	208 (28.65%)	
Maternal age (mean (SD), years)	30.07 (4.42)	30.99 (5.29)	0.02^b^
BMI (mean (SD), kg/m^2^)	21.03 (2.95)	21.14 (2.55)	0.73^b^
Smoking history (*N* (%))	2 (0.39%)	0 (0.00%)	0.37^a^
Drinking history (*N* (%))	1 (0.19%)	0 (0.00%)	0.53^a^
Gestational week (mean (SD))	6.24 (1.67)	6.32 (1.74)	0.57^b^
Gestity (medium (IOR), times)	2.00 (2.00-3.00)	3.00 (2.00-4.00)	<0.05^c^
Parity (medium (IOR), times)	0.00 (0.00-1.00)	0.00 (0.00-1.00)	0.06^c^
Abortion (medium (IOR), times)	1.00 (0.00-2.00)	1.00 (0.00-2.00)	0.66^c^
Progesterone^▲^ (medium (IOR), nmol/L)	89.91 (66.86-123.00)	67.59 (39.47-95.42)	<0.01^c^
*β*-HCG^▲^ (log10) (mean (SD))	4.25 (0.82)	3.64 (0.81)	<0.01^b^
Embryo visualized (*N* (%))	328 (63.32%)	56 (27.05%)	<0.01^a^
Cardiac movements visualized (*N* (%))	323 (62.36%)	38 (18.45%)	<0.01^a^
Marital status			0.74^a^
Unmarried (*N* (%))	12 (2.32%)	4 (1.92%)	
Married (*N* (%))	505 (97.68%)	204 (98.08%)	
Medical history			
Diabetes (*N* (%))	2 (0.39%)	2 (0.96%)	0.34^a^
Systemic lupus erythematosus (*N* (%))	1 (0.19%)	1 (0.48%)	0.50^a^
Polycystic ovary syndrome (*N* (%))	25 (4.83%)	5 (2.40%)	0.14^a^
Endometriosis (*N* (%))	35 (6.76%)	16 (7.69%)	0.66^a^
Hyperprolactinemia (*N* (%))	9 (1.74%)	4 (1.92%)	0.87^a^
Using progesterone (*N* (%))	405 (78.19%)	162 (77.88%)	0.93^a^

^▲^Basal progesterone and basal *β*-HCG were reported. ^a^We used a chi-square test to analyse the data of smoking history, drinking history, embryo visualization, cardiac movement visualization, marital status, diabetes, systemic lupus erythematosus, polycystic ovary syndrome, endometriosis, hyperprolactinemia, and whether using progesterone. ^b^We used the Student *t*-test to analyse the data of maternal age, BMI, gestational week, and basal *β*-HCG (log10). ^c^We used the Mann-Whitney *U* test to analyse the data of gestity, parity, abortion, and basal serum progesterone.

**Table 2:** Results of univariate multivariate analysis using binary logistic regression and marginal structural model.

Exposure	Model 1: nonadjusted model (OR, 95% CI, *P* value)	Model 2: fully adjusted model (OR, 95% CI, *P* value)	Model 3: marginal structural model (HR, 95% CI, *P* value)
Progesterone	0.98 (0.98, 0.99) <0.01	0.99 (0.98, 0.99) <0.01	0.99 (0.98, 0.99) <0.01
Progesterone tertile			
Low	Ref	Ref	Ref
Middle	0.40 (0.27, 0.59) <0.01	0.47 (0.24, 0.89) <0.05	0.45 (0.19, 1.07) <0.01
High	0.28 (0.18, 0.42) <0.01	0.48 (0.25, 0.92) <0.05	0.35 (0.18, 0.71) <0.01
*P* for trend of	<0.01	<0.05	<0.01

Nonadjusted model: we used basal progesterone, and no covariables were adjusted. Fully adjusted model: we used basal progesterone and adjusted for maternal age, BMI, smoking, drinking, gestational week, marital status, basal *β*-HCG (log10), gestity, parity, abortion, embryo visualization, cardiac movement visualization, medical history, and use of progesterone. Marginal structural model: we used serial progesterone and adjusted for maternal age, BMI, smoking, drinking, gestational week, marital status, serial *β*-HCG (log10), gestity, parity, abortion, embryo visualization, cardiac movement visualization, medical history, and use of progesterone.

**Table 3:** Nonlinearity addressing of progesterone.

Exposure	Progesterone (OR, 95% CI, *P* value)
Fitting model using standard binary logistic regression model	0.99 (0.98, 0.99) <0.01
Fitting model using two-piecewise regression model	
Inflection point	90.62
<inflection point	0.97 (0.95, 0.98) <0.01
≥inflection point	1.01 (0.99, 1.02) 0.27
*P* for log-likelihood ratio test	<0.01

We used basal progesterone and adjusted for maternal age, BMI, smoking, drinking, gestational week, marital status, basal *β*-HCG (log10), gestity, parity, abortion, embryo visualization, cardiac movement visualization, medical history, and use of progesterone.

## Supplementary Materials

Supplementary Materials Both progesterone and *β*-HCG change over time. The supplementary material (Schedules 1-6) describes the trend of progesterone and *β*-HCG in different groups. In the schedule, the abscissa indicates the times of measurements and the ordinate indicates the mean and 95% CI of progesterone or *β*-HCG. Schedules 1-3 are about progesterone in nmol/L, while schedules 4-6 are about *β*-HCG in IU/L. Schedule 1 shows the trend of progesterone of threatened abortion patients. Schedule 2 shows the trend of progesterone of participants who continued pregnancy after 12 weeks of gestation. Schedule 3 shows the trend of progesterone of participants who miscarriage within 12 weeks. Schedule 4 shows the trend of *β*-HCG of threatened abortion patients. Schedule 5 shows the trend of *β*-HCG of participants who continued pregnancy after 12 weeks of gestation. Schedule 6 shows the trend of *β*-HCG of participants who miscarriage within 12 weeks.
