# Supplementary material for: Baseline Levels of Serum Progesterone and the First Trimester Pregnancy Outcome in Women with Threatened Abortion: A Retrospective Cohort Study
Source: Biomed Res Int. 2020 Mar 2;2020:8780253. doi: 10.1155/2020/8780253 (PMC7072098; doi:10.1155/2020/8780253)

Schedule 1: the trend of progesterone over times of measurements in threatened abortion patients

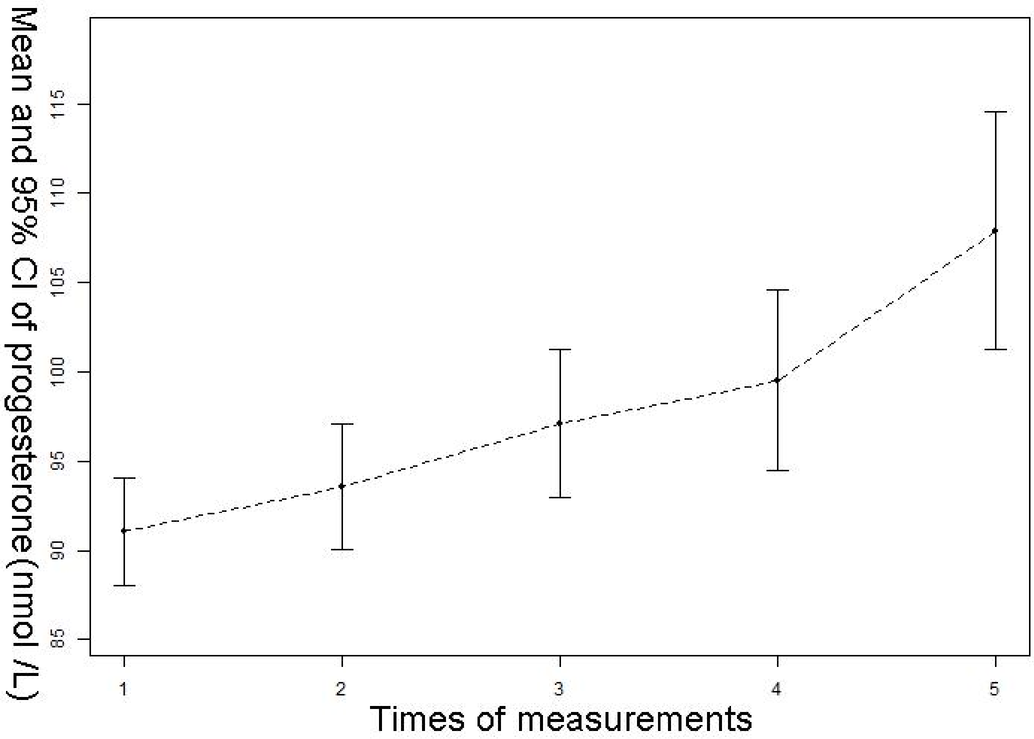

Schedule 2: the trend of progesterone over times of measurements in threatened abortion patients who continued pregnancy

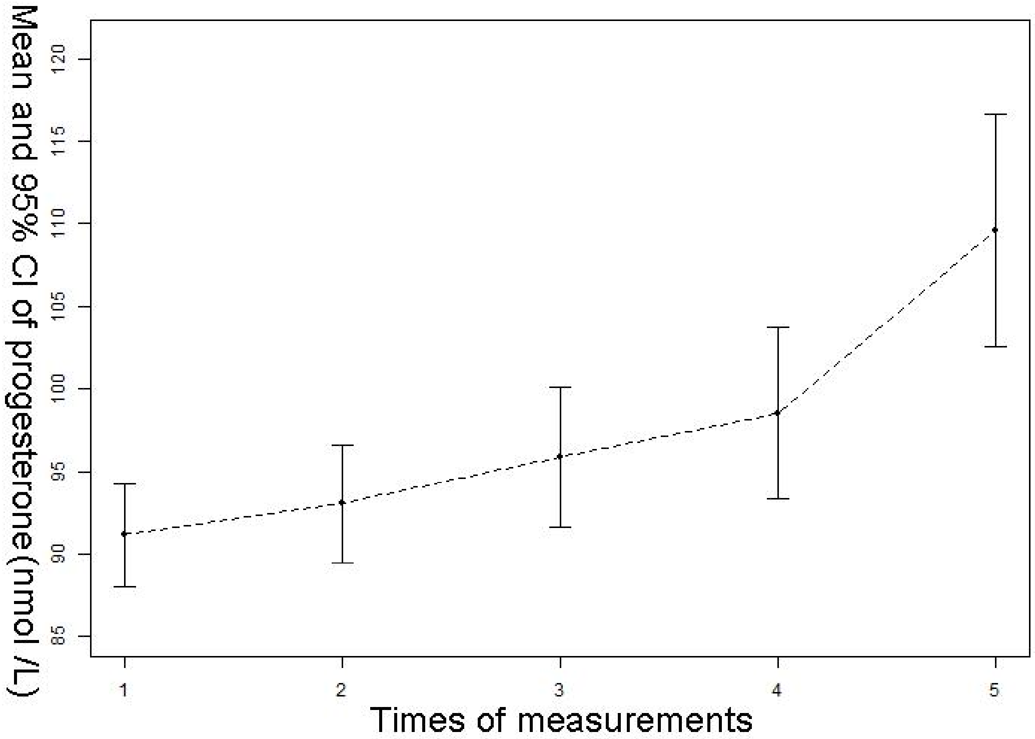

Schedule 3: the trend of progesterone over times of measurements in threatened abortion patients who miscarriage within 12 weeks

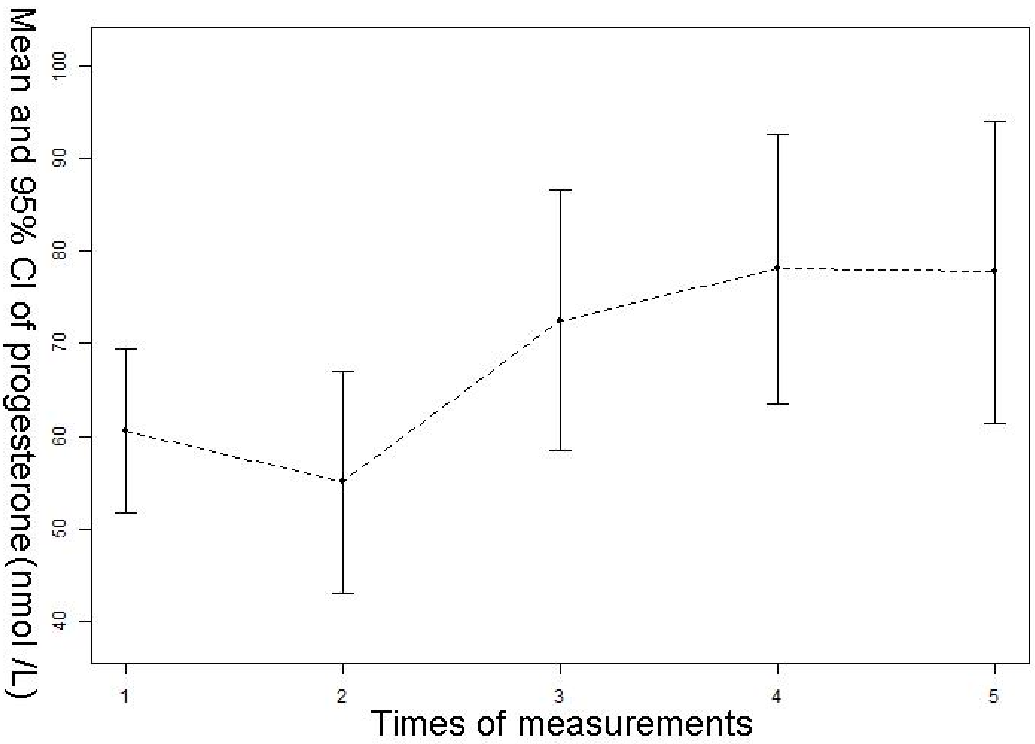

Schedule 4: the trend of β-HCG over times of measurements in threatened abortion patients

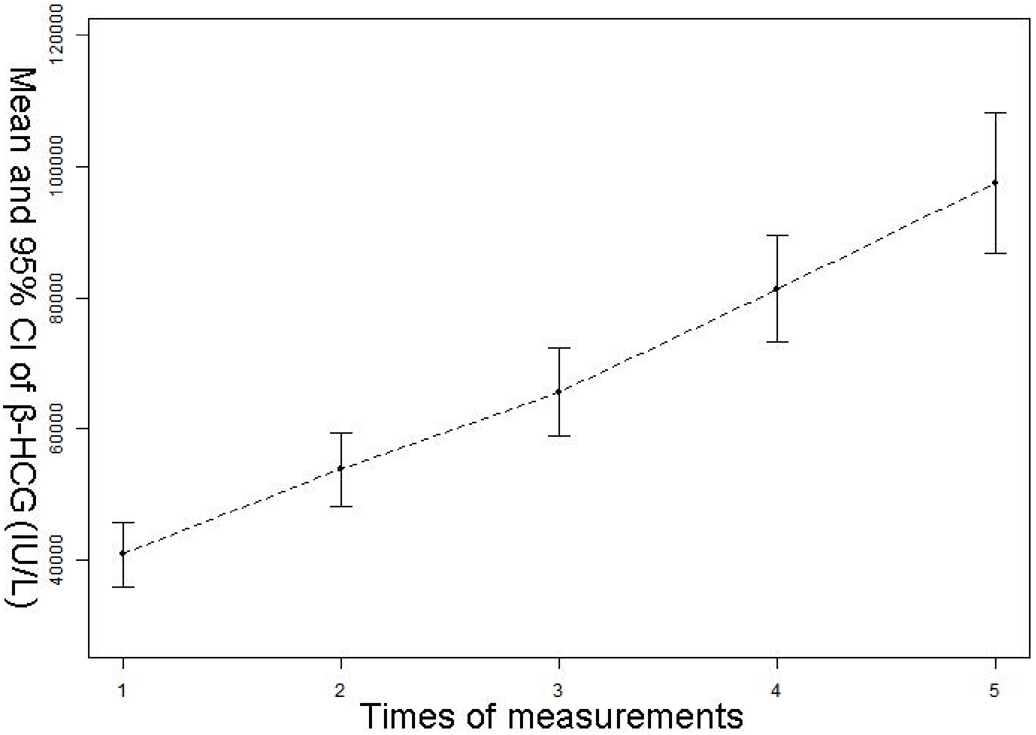

Schedule 5: the trend of β-HCG over times of measurements in threatened abortion patients who continued pregnancy

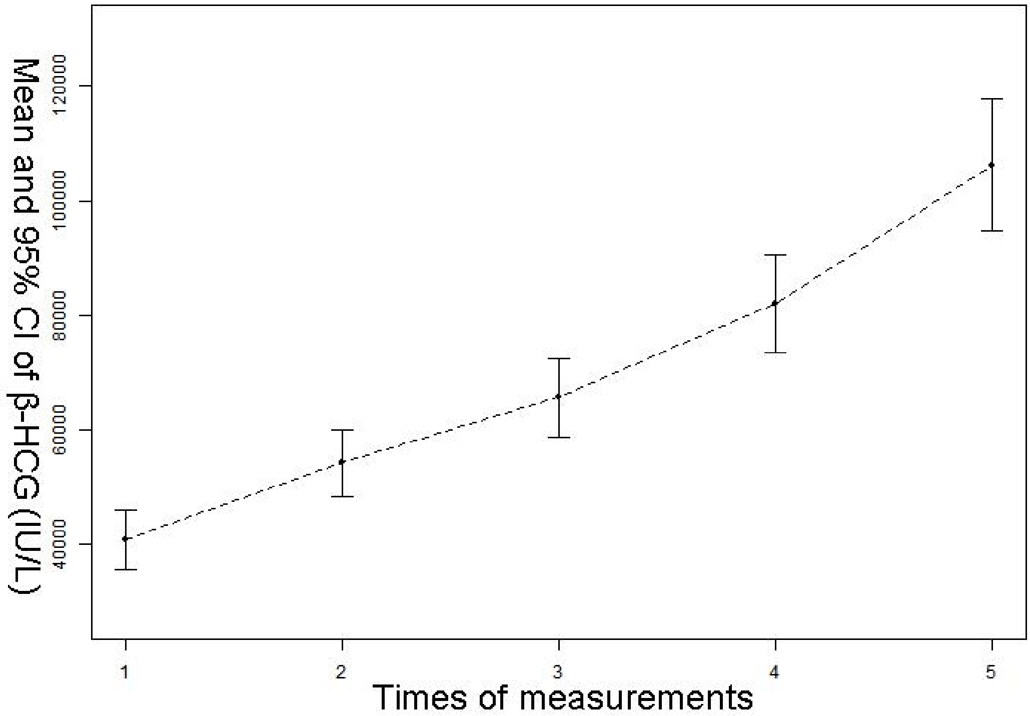

Schedule 6: the trend of β-HCG over times of measurements in threatened abortion patients who miscarriage within 12 weeks

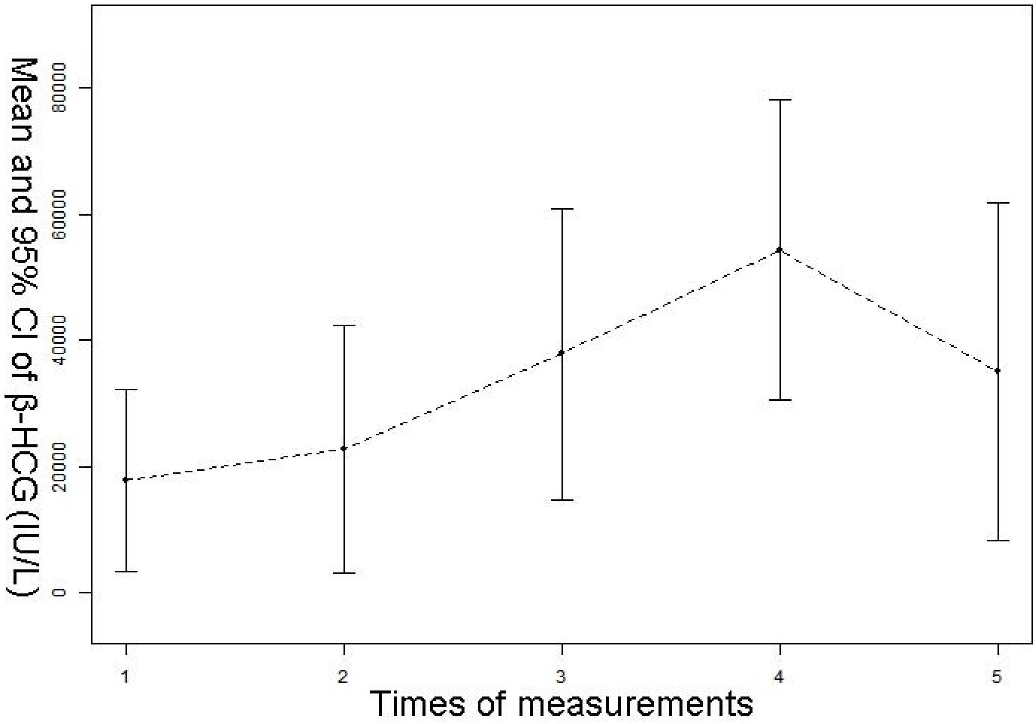

Supplement: Supplementary Materials — Both progesterone and β-HCG change over time. The supplementary material (Schedules 1-6) describes the trend of progesterone and β-HCG in different groups. In the schedule, the abscissa indicates the times of measurements and the ordinate indicates the mean and 95% CI of progesterone or β-HCG. Schedules 1-3 are about progesterone in nmol/L, while schedules 4-6 are about β-HCG in IU/L. Schedule 1 shows the trend of progesterone of threatened abortion patients. Schedule 2 shows the trend of progesterone of participants who continued pregnancy after 12 weeks of gestation. Schedule 3 shows the trend of progesterone of participants who miscarriage within 12 weeks. Schedule 4 shows the trend of β-HCG of threatened abortion patients. Schedule 5 shows the trend of β-HCG of participants who continued pregnancy after 12 weeks of gestation. Schedule 6 shows the trend of β-HCG of participants who miscarriage within 12 weeks. [file 8780253.f1.pdf]
